# Exploitative Competition between Mountain Hare and Moose—Qualitative Effects on Hare Winter Forage?

**DOI:** 10.3390/ani11092638

**Published:** 2021-09-08

**Authors:** Simen Pedersen, Hans Chr. Pedersen

**Affiliations:** 1 Faculty of Applied Ecology, Agricultural Sciences and Biotechnology, Campus Evenstad, Inland Norway University of Applied Sciences, N-2480 Koppang, Norway; 2 Norwegian Institute for Nature Research, Høgskoleringen 9, N-7485 Trondheim, Norway; hans.pedersen@nina.no

**Keywords:** browsing, cafeteria test, large herbivore, hind gut fermenter, ruminant, lagomorph, cervid

## Abstract

**Simple Summary:**

Mountain hares in Scandinavia are classified as Near Threatened in the Norwegian and Swedish Redlists assessing the risk of species extinction. This is due to a possible population decline witnessed during the last decades in Scandinavia. Competition between large herbivores such as moose, red deer, roe deer on one hand and hares on the other, is one of several hypotheses that has been put forward to explain this decline. In a cafeteria trial (providing several types of forage to determine food preference) we investigate the effects of previous moose winter foraging on the food selection (i.e., amount consumed, bites per minute and bitediameter) of downy birch and goat willow by captive hares. We find that hares do not differentiate among levels of previous moose foraging on downy birch but have larger bite diameters of goat willow earlier eaten on by moose, compared to plants not fed on by moose. Thus, effects of moose on hare winter food quality seem to be limited. We highlight the need for studies focusing on (1) effects of previous moose foraging using wild hares in a natural experimental design, and (2) effects of moose foraging on available hare food at a landscape scale during winter.

**Abstract:**

Mountain hares (*Lepus timidus*) in Scandinavia are classified as *Near Threatened* in the Norwegian and Swedish Redlists. This is due to a possible population decline witnessed during the last decades in Scandinavia. Competition between large herbivores and mountain hares is one of several hypotheses that has been put forward to explain this decline. In a cafeteria trial we investigate the effects of previous moose (*Alces alces*) winter browsing on the food selection (i.e., biomass consumed, bites per minute and bitediameter) of downy birch (*Betula pubescens*) and goat willow (*Salix caprea*) by captive mountain hares. We find that mountain hares do not differentiate among previous browsing levels of downy birch, but have larger bite diameters of goat willow earlier browsed by moose, compared to non-browsed plants. Thus, effects of moose on mountain hare winter food quality seem to be limited. We highlight the need for studies focusing on (1) qualitative effects of moose browsing using wild mountain hares in a natural experimental design, and (2) quantitative effects of moose browsing on available mountain hare forage at a landscape scale during winter.

## 1. Introduction

In Scandinavia, mountain hares (*Lepus timidus*), have shown possible population declines over the past decades [1,2,3] (www.viltdata.se accessed on: 27 August 2021) and were classified as *Near Threatened* in the Norwegian Redlist in 2015 and again in 2021 (www.artsdatabanken.no accessed on: 27 August 2021), as well as the Swedish Redlist in 2020 (www.artfakta.se accessed on: 27 August 2021). Several hypotheses have been put forward to explain this decline including climate change, land use change, parasites, predation, and competition [3]. Climate change, color mismatch and increased predation is currently a topic of general research interest, and we have earlier shown that abundance of mountain hares is negatively affected by an interaction between reduced snow cover duration and abundance of generalist predators; red fox (*Vulpes vulpes*) and pine marten (*Martes martes*) [1]. However, the mountain hare may not only be negatively affected by top-down effects, but also bottom-up through interactions with other species from within the herbivore guild [4]. On a landscape scale, moose (*Alces alces*) is known to reduce abundance of deciduous trees e.g., [5,6], and mountain hares are negatively associated with moose presence at a habitat patch scale [7]. Thus, in areas of high density, moose may be an important competitor towards mountain hares [8].

Herbivore species are not expected to compete if they either differ in digestive systems [9], or in body size [10]. Hence, one would expect moose and mountain hares to have large ecological niche separation in terms of diet overlap and potential for food competition. However, moose [11] and mountain hare [12] consume several of the same plant species (i.e., birch (*Betula* spp., salix (*Salix* spp.) and aspen (*Populus tremula*), especially during winter. Herbivore species’ food preferences vary along a gradient from bulk feeding of low nutritious forage to selective feeding of high-quality forage see [13], and references therein. On one hand, hindgut fermenters (such as the mountain hare) have less efficient digestive system compared to ruminants (such as the moose). Due to their relatively inefficient digestive systems, hind gut fermenters select plant quantity over quality. Small herbivores on the other hand, are in general more selective, targeting higher food quality compared to large-bodied herbivores [13]. Ironically, due to this interaction between body size and digestive systems—a large ruminant and a small hindgut fermenter may compete over food sources. Indeed, previous studies have shown mountain hares to be competitively inferior to larger herbivores such as the roe deer (*Capreolus capreolus*) [14]. Hulbert and Andersen [14] did not detect any feeding-height separation between the two species, however mountain hares switched to smaller bite diameters when in sympatry with roe deer, likely leading to higher concentrations of harmful plant secondary metabolites. The authors suggest that this switch may lead to reduced survival of mountain hares in the presence of roe deer. Large herbivores such as moose and roe deer may therefore limit mountain hare densities, especially in areas of high cervid density. These negative impacts may either be transferred through reduced forage quantity, i.e., deciduous winter browse [5,6] or forage quality through chemical changes in plants [14,15].

To investigate the latter competitive pathway of reduced food quality we conducted cafeteria trials with captive mountain hares, using downy birch (*Betula pubescens*) and goat willow (*Salix caprea*), two species which are consumed by moose and mountain hares in winter. For both moose and mountain hares, downy birch is the staple food, while goat willow is among the highly preferred plant species [11,12,15,16]. We predicted that mountain hares would prefer unbrowsed downy birch due to a lower concentration of plant secondary metabolites [17]. For goat willow we predicted that mountain hares would prefer previously browsed shoots, as the related tea-leaved willow (*S. phylicifolia*) is known to produce shoots with higher biomass and lower levels of chemical defense as a response to moose browsing [18].

## 2. Methods

### 2.1. Sample Collection

This study was carried out in Østerdalen (61° N, 11° E), Southeast Norway along a gradient in moose density and browsing pressure. In January 2008, we collected downy birch and goat willow branches (approximately 30–40 cm long), after inset of winter dormancy but without signs of recent browsing. We here consider branches consisting of several years’ growth, while shoots are one year’s growth. All branches were collected from trees <2.5 m height. Any collected shoots with signs of current moose browsing were discarded. Shoot coloration and bark structure may be used to separate the most current growing season from previous years growing seasons. Browsing levels were estimated as a percentage of available shoots browsed during the previous browsing season. For downy birch, we collected branches without (0%), intermediate (30% < 60%), and high levels of previous moose browsing (>90%). We also collected goat willow without browsing (0%) and more than 30% previously browsed shoots. Browsing levels were determined based on morphology of the branches. After browsing, lateral shoots tend to form leaving a dry «stump» where the moose bit off the shoot last winter. We specifically targeted the mentioned percentage intervals as to avoid overlapping of categories—thus, this approach should be robust towards any slight over or underestimation of percentage browsing. We originally aimed for three categories of goat willow, like that of downy birch, but high moose densities and limited availability of goat willow prevented us from doing so. For the control (no browsing) we collected branches from two military facilities (Mil I and Mil II) within the Østerdalen valley, which were fenced more than 30 years before the experiment. The other treatments were collected just outside the two military facilities as well as two additional areas (Imsdalen and Kopppangskjølen) known to have high moose presence and browsing pressure in winter [19,20]. All four sites lie within the boreal forest and have similar forest composition dominated by Norway spruce (*Picea abies),* Scots pine (*Pinus sylvestris*), interspersed with downy birch, silver birch (*B. pendula*), *Salix.* spp., aspen, rowan (*Sorbus aucuparia*) and grey alder (*Alnus incana*). Samples were collected at these four sites depending on availability. Thus, for Imsdalen we had the categories “willow browsed”, “birch intermediate”, and “birch high”. For Koppangskjølen we had “birch intermediate” and “birch high”, For Mil I we had “birch no browsing”, “willow no browsing”, “birch intermediate” and “willow browsed”. For Mil II we collected “birch no browsing”, “willow no browsing”, and “willow browsed”.

### 2.2. Cafeteria Test

Fourteen captive mountain hares were housed in cages with ad lib. access to water, rabbit pellets, and branches from various deciduous trees. The mountain hares were part of a breeding facility for restocking natural populations. No permit was required under the Norwegian Food Safety Authority as the experiment was part of the natural feeding of the animals. Mountain hares were kept indoors in an unheated barn. For two days prior to the start of the experiment, mountain hares were fed downy birch and goat willow, in order to habituate them to the material to be used in the experiment.

Mountain hares were deprived of food (12 h) over the night before the experiment started. We provided the mountain hares with bundles of branches, weighing approximately 100 g (±0.01 g), for 90 min. All 14 mountain hares were given all five categories/species of branches in randomized order, over a six-day period. Immediately after finishing the trial, we measured bite diameter and shoot base diameters (to the nearest 0.1 mm) of the remaining material using callipers. Bundles and all shoot residues on the cage floor were weighed and we calculated wet biomass consumed. We weighed control bundles of goat willow and downy birch not used in the experiment but treated similarly. Mean weight loss due to evaporation was 0.10 g and 0.2 %; thus, we regarded all weight loss as biomass consumed.

Additionally, mountain hares were filmed during the cafeteria trial, and we registered time spent feeding, and number of bites. Some of the recordings failed to document all mountain hare activity during the trial and were thus excluded from further analysis.

### 2.3. Statistical Analysis

The dataset consisted of branches from four different areas, the means of response variables varied among the different areas (Table 1); however, the 95% CI overlapped for tree species as well as browsing levels. Thus, these differences were not considered for further analysis, but our results should reflect general patterns as the material was collected from several sites.

For the two species goat willow and downy birch we ran separate mixed effects ANOVAs with grams consumed, bites per minute and bitediameter as response variables, and moose browsing intensity as explanatory variable. We included mountain hare individual as a random term, to account for individual variation among mountain hares. Analyses were performed in R version 4.0.4 [21].

## 3. Results

**Figure 1:**
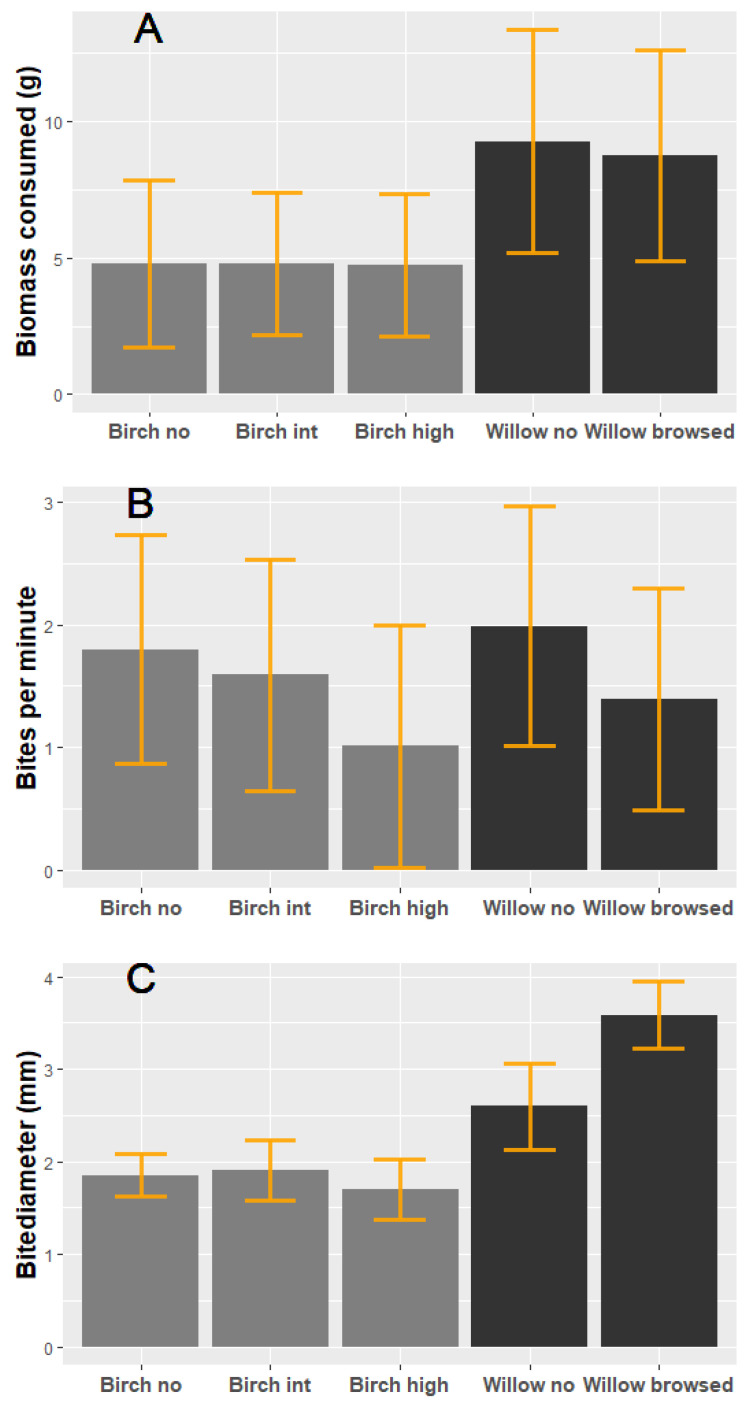
(**A**) consumption, (**B**) bites per minute and (**C**) bitediameter of downy birch (light bars) and goat willow (dark bars) ±95% CI in relation to previous moose browsing. Birch no (0% browsed), Birch int (30% < 60% browsed), and Birch high (>90% browsed). Willow no (0% browsing) and Willow browsed (30% < browsed). Estimates from a mixed effects ANOVA with mountain hare individual as a random term. Note that goat willow and downy birch are run in different models due to differences in browsing categories but plotted together.

For downy birch we found no effects on amount consumed (Figure 1A), bites per minute (Figure 1B) or bite diameter (Figure 1C); thus, mountain hares did not differentiate among level of previous browsing from moose (Table 2). For goat willow we only found an effect of treatment on bite diameter (Figure 1C), with mountain hares having larger bite diameter for goat willow previously browsed by moose (Table 2). For the variables biomass consumed (Figure 1A) and bites per minute (Figure 1B) we found no effect of treatment (Table 2). Overall, biomass of goat willow consumed was higher than that of downy birch (Figure 1A).

## 4. Discussion

For downy birch we found no effect of previous moose browsing on mountain hare preference. In a similar field-based study by Danell and Huss-Danell [17], mountain hares also did not differentiate between moderate and low previous moose browsing on birches (*B. pubescens* and *B. pendula*). For goat willow we found an effect on bite diameter only, where mountain hares have larger bite diameters on goat willow previously browsed by moose. Herbivores are known to select for large shoot base diameters [22], and our results corresponds to findings by Stolter (2008) in the closely related tea-leaved willow that is known to respond to moose winter browsing by producing thick shoots, which again is preferred by moose the following winter.

Plants may respond to browsing by tolerance or avoidance. Avoidance of herbivory may either be escaping browsing altogether or physical and chemical defense [23]. Chemical defense may be constitutive (i.e., omnipresent) or induced as a response to damage by e.g., herbivores [23,24]. Regrowth after moose browsing on the tea-leaved willow produced shoots with lower concentration of plant secondary compounds (Stolter 2008). This counter-intuitive response led to a positive feedback loop, where moose rebrowsed tea-leaved-willow that were browsed during the previous browsing season (Stolter 2008). Although we did not investigate chemical composition in the treatments, it is possible that such a positive feedback loop with decreased levels of plant secondary compounds following moose browsing is the case also with goat willow in the current study.

The lack of a strong effect of previous moose browsing in the current study could be due to several reasons: (1) The mountain hares used in the experiment are captive; thus, their food selection and preferences may not be reflecting that of wild mountain hares. (2) Many plant secondary compounds act as digestive inhibitors e.g., [25]. Thus, if the mountain hares of our study have unlimited access to high quality rabbit pellets, any response in the plants to moose browsing leading to increased concentration of digestive inhibitors may be swamped by the mountain hares’ unlimited access to high quality food.

## 5. Conclusions

In recent years, several studies have suggested competition between lagomorphs and cervids. In Italy, European hare (*L. europaeus*) and roe deer are known to increase their diet overlap in winter [26]; however, they seem to reduce the degree of competition by having low spatial overlap on the landscape scale [27], similarly to that of moose and mountain hares in Northern Sweden [7].

From our results and those of others [17] we suggest that if there is a competitive relationship between moose and mountain hares, this is not likely caused by qualitative changes in mountain hare winter forage, but rather quantitative changes as a result of moose reducing available mountain hare winter forage in the landscape. Future studies should investigate (1) any qualitative effects (i.e., impacts on plant secondary compounds) of moose browsing using wild mountain hares in a natural experimental design, and (2) the possible alternative competitive route of quantitative effects of moose on available mountain hare forage at a landscape scale during winter.

## Figures and Tables

**Table 1:** Mean values (±95 % CI) of biomass consumed, bites per minute, bitediameter (mm) and shoot basediameter (mm) for downy birch without (0%), intermediate (30% < 60%), and high levels of previous moose browsing (>90%), and goat willow without browsing (0%) and more than 30% previously browsed. Branches were collected in the four areas Imsdalen, Koppangskjølen Military area I and II.

Area	Material	Consumed (g)	Bites per min	Bitediam	Basediam
Imsdalen	*Birch intermediate*	4.79 (5.30)	1.25 (1.73)	2.08 (0.66)	2.41 (0.97)
	*Birch high*	5.58 (4.02)	0.41 (0.80)	1.60 (0.90)	2.66 (0.94)
Koppang	*Willow browsed*	8.47 (2.77)	1.80 (0.62)	4.02 (0.69)	7.11 (3.34)
	*Birch intermediate*	3.03 (4.08)	2.07 (1.80)	1.82 (1.05)	2.84 (1.20)
	*Birch high*	3.91 (4.78)	1.03 (1.02)	1.72 (0.53)	2.60 (0.56)
Mil I	*Birch no*	3.81 (2.29)	1.90 (1.70)	1.99 (0.58)	2.52 (0.70)
	*Birch intermediate*	6.48 (0.90)	1.38 (0.38)	1.91 (0.29)	2.61 (0.38)
	*Willow no*	9.94 (5.61)	3.15 (1.94)	2.45 (0.45)	3.25 (0.76)
	*Willow browsed*	11.94 (5.53)	0.95 (0.11)	3.20 (0.81)	4.91 (1.41)
Mil II	*Birch no*	5.77 (6.88)	1.60 (1.54)	1.68 (0.43)	2.41 (0.59)
	*Willow no*	8.62 (3.35)	1.09 (1.28)	2.70 (0.08)	3.56 (1.19)
	*Willow browsed*	5.92 (3.11)	1.02 (1.14)	3.39 (2.33)	5.81 (6.93)

**Table 2:** Results from Mixed effects ANOVAS with treatment as explanatory variable and mountain hare individual as a random term.

Species	Variable	*n*	df	*F*	*p*
Downy birch	*Biomass consumed*	30	2, 18	<0.001	0.999
	*Bites per minute*	22	2, 12	1.400	0.285
	*Bitediameter*	27	2, 15	0.872	0.438
Goat willow	*Biomass consumed*	20	1, 9	0.068	0.801
	*Bites per minute*	15	1, 6	1.497	0.267
	*Bitediameter*	18	1, 7	5.194	0.048

## Data Availability

The data presented in this study are available on request from the corresponding author.
